# A Model for Changes in Germination Synchrony and Its Implements to Study Weed Population Dynamics: A Case Study of Brassicaceae

**DOI:** 10.3390/plants12020233

**Published:** 2023-01-04

**Authors:** Keyvan Maleki, Kourosh Maleki, Elias Soltani, Mostafa Oveisi, Jose L. Gonzalez-Andujar

**Affiliations:** 1 Department of Horticulture and Crop Science, Ohio State University, Columbus, OH 43210, USA; 2 Department of Forestry, Gorgan University of Agricultural Sciences and Natural Resources, Gorgan P.O. Box 386, Iran; 3 Department of Agronomy and Plant Breeding Sciences, College of Aburaihan, University of Tehran, Tehran 1417466191, Iran; 4 Department of Agronomy and Plant Breeding, College of Agriculture & Natural Resources, University of Tehran, Karaj 1417466191, Iran; 5 Instituto de Agricultura Sostenible (CSIC), 14004 Córdoba, Spain

**Keywords:** dormancy continuum, conditional dormancy, physiological dormancy, synchrony pattern, weed population dynamics

## Abstract

In every agricultural system, weed seeds can be found in every cubic centimeter of soil. Weed seeds, as a valuable trait underlying the fate of weed populations, exhibit differing levels of seed dormancy, ensuring their survival under uncertain conditions. Seed dormancy is considered as an innate mechanism that constrains germination under suitable conditions that would otherwise stimulate germination of nondormant seeds. This work provides new insight into changes in germination patterns along the dormant to nondormancy continuum in seeds with physiological dormancy. Notable findings are: (1) germination synchrony can act as a new parameter that quantitatively describes dormancy patterns and, subsequently, weed population dynamics, (2) germination synchrony is dynamic, suggesting that the more dormancy decreases, the more synchrony is obtainable, (3) after-ripening and stratification can function as a synchronizing agent that regulates germination behavior. Freshly harvested seeds of *Brassica napus* with type 3 of non-deep physiological dormancy showed the most synchronous germination, with a value of 3.14, while a lower level of germination asynchrony was found for newly harvested seeds of *Sinapis arvensis* with type 1 of non-deep physiological dormancy, with an asynchrony value of 2.25. After-ripening and stratification can act as a synchronizing factor through decreasing the asynchrony level and increasing synchrony. There is a firm relationship between seed dormancy cycling and germination synchrony patterns, ensuring their survival and reproductive strategies. By germinating in synchrony, which is accompanied by cycling mechanisms, weeds have more opportunities to persist. The synchrony model used in the present study predicts germination behavior and synchrony along the dormant to nondormancy continuum in weed seeds with physiological dormancy, suggesting a useful method for the quantification of germination strategies and weed population dynamics.

## 1. Introduction

The existence of large weed seedbanks in agricultural soils has roots in the seed production of the past, leading to weed invasion in the current year and in not-so-distant future years [1]. Weed seeds, as a valuable trait underlying the fate of weed populations, exhibit different levels of seed dormancy, ensuring their survival under uncertain conditions.

Seed dormancy is considered as an innate mechanism that constrains germination under suitable conditions that would otherwise stimulate the germination of nondormant seeds [2]. However, quiescence is defined as the inability to germinate as a result of lacking suitable environmental conditions required for germination [2]. Dormancy patterns allow seeds to avoid absolute germination during short spells of ephemerally favorable environments, in which germination may not be successful [1,2]. Germination, as a critical stage in plant life history, plays a vital role in establishing plants and determining suitable conditions in which other life-history events can take place [2,3]. Seed germination and seedling emergence, regarded as the most sensitive phases to environmental stochasticity, can influence individual fitness, population persistence and the distribution of species [3,4,5,6,7,8]. Generally, dormancy patterns allow species to establish appropriate mechanisms, in which species make a choice of habitat and construct a germination niche in response to the environmental conditions they experience [9].

Soil seedbanks store viable and non-germinated (dormant) weed seeds to maximize survival and reproduction in the face of environmental hazards. The soil seedbank has various implications for weed population dynamics, particularly through maintaining adequate insurance for population persistence and populations occupying habitats exposed to stochasticity [1,2]. Environmental conditions may impede germination from the seedbank so that germination and seedling emergence can occur when conditions are suitable for germination and seedling emergence, thereby promoting survival after dispersal. Thus, the primary role of seedbanks is to maintain seed viability and persistence until the environmental conditions best for recruitment arrive [2].

The soil seedbank may function as a buffer against annual variation in environmental conditions. In environments with erratic rainfall, natural selection will favor higher dormancy fraction. In contrast, a low level of dormancy is favored in predictable environments, in which a high frequency of rainfall is likely to occur [10]. Nevertheless, seed viability in the soil seedbank is directly influenced by seed dormancy [2]. Many plants may have specific thermal and moisture requirements for germination, and global climate change may pose the risk of unfulfillment of the requirements [2,11]. In addition, germination timing, germination success, seed persistence and dormancy breakage are considerably influenced by environmental cues [12,13,14,15,16,17]. Additionally, germination traits that enable species to be responsive to environmental cues can result in the evolution of germination requirement, thereby influencing population dynamics [18].

Different classifications of seed dormancy have been proposed [19,20] but the system developed by Nikolaeva seems the most informative classification [2]. Three levels of physiological dormancy are identified, namely nondeep, intermediate and deep [2]. It has been proven that different periods of warm and cold stratification, after-ripening and Gibberellic acid (GA_3_) can break non-deep physiological dormancy [2,16,21]. Various methods have been considered as a model for changes in dormancy level. In considering the breakage of non-deep physiological dormancy, much attention has been devoted to developing quantitative approaches that describe changes in dormancy status and germination patterns [16,21,22]. Studies have reported that temperature range permissive for germination widened as dormancy level changed [16], while other studies showed that the values of base water potential can identify dormancy level and germination patterns [2,23,24]. Moreover, a recent study on germination of an annual species indicated that plant species maintain seed dormancy to control germination synchrony and time germination with suitable conditions required for germination [25].

Given the importance of seed dormancy and quantitative approaches for describing the dormancy level in seeds with physiological dormancy, various models and parameters that are ecologically meaningful have been developed to describe dormancy status and changes in dormancy level in seeds undergoing dormancy-breaking treatments [2,16,25]. Hydrotime and hydrothermal time models are well-established statistical tools used for quantification of seed germination of weeds [2,23,24].

In the current work, we propose a new conceptual framework for quantifying dormancy status and germination behavior, which has ecologically meaningful parameters describing the relationship between seedbank dynamics, germination synchrony and dormancy status. In contrast to other models used for this purpose, such as hydrotime and hydrothermal time, the proposed model enables researchers and weeds scientists to understand dormancy dynamics in the soil seedbank and the way in which seed dormancy regulates weed population dynamics. We sought to address the following questions: (1) Does dormancy level underlie germination synchrony patterns of weed populations? (2) Do synchrony patterns affect weed population dynamics in the soil seedbank? (3) Does dormancy type play a role in shaping germination synchrony? (4) Are after-ripening and stratification synchronizing agents? (5) Is synchronization pattern dynamic? To address the above-mentioned issues, two populations of Brassicaceae family, which are common in arable lands, were considered. We aimed to show that a population-based model can simply illuminate how the population dynamics of weeds can be affected by dormancy and synchrony.

## 2. Results

### 2.1. Dynamic Synchronization

**Figure 1:**
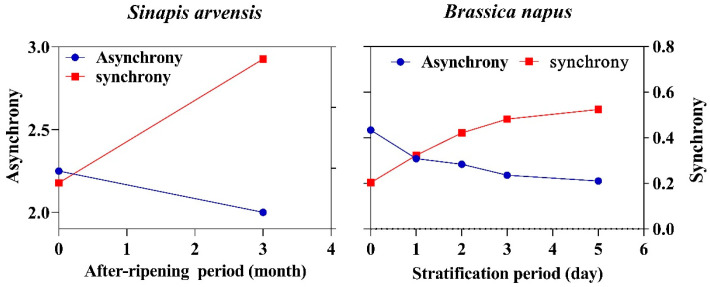
The changes in synchrony and asynchrony patterns in response to after-ripening and stratification periods.

Under the assumption of synchronization process, seeds of species with non-deep physiological dormancy may progressively gain the ability to germinate in concert the more dormancy terminates. Then, we developed a new conceptual framework for understanding changes in germination synchrony along the dormant to nondormancy continuum, so-called dynamic synchronization. To test the above assumption, we used two dormancy-breaking agents (after-ripening and stratification) applied to two weeds with non-deep physiological dormancy to see how seeds with varying dormancy level can undergo the synchronization process. An inverse relationship was observed between germination synchrony and asynchrony in seeds going through dormancy-breaking agents. Both after-ripening and stratification enhanced germination synchrony (Figure 1). Seeds of *Sinapis arvensis* progressively became synchronized as they experienced after-ripening for three months. Those seeds that experienced three months of after-ripening showed more synchronous germination than mature seeds, while the highest level of asynchrony was observed in mature seeds (Figure 1). Stratification changed synchrony patterns as after-ripening did, suggesting that various periods of stratification may have synchronizing effects on germination patterns. The most synchronous and asynchronous germination was observed for seeds stratified for 5 days and mature seeds of *B. napus* (Figure 1).

### 2.2. Germination Synchrony Patterns

Based on the results from the empirical experiments, germination synchrony directly corresponded with the level of dormancy. Germination asynchrony was different between two species, ranging from 3.14 in *B. napus* to 2.25 in *S. arvensis*. Since application of stratification and after-ripening enabled seeds to germinate in synchrony (higher value of germination synchrony versus lower value of germination asynchrony; Table 1), dormancy-breaking agents can be considered as a synchronizing factor, which acts in an efficient way. The highest synchronous value for germination was observed for *S. arvensis*, with a value of 0.14, while the rate of synchronicity in the other species was slow and it progressively gained the ability to germinate in synchrony (Table 1).

### 2.3. Dormancy Cycling and Germination Synchrony Patterns

**Figure 2:**
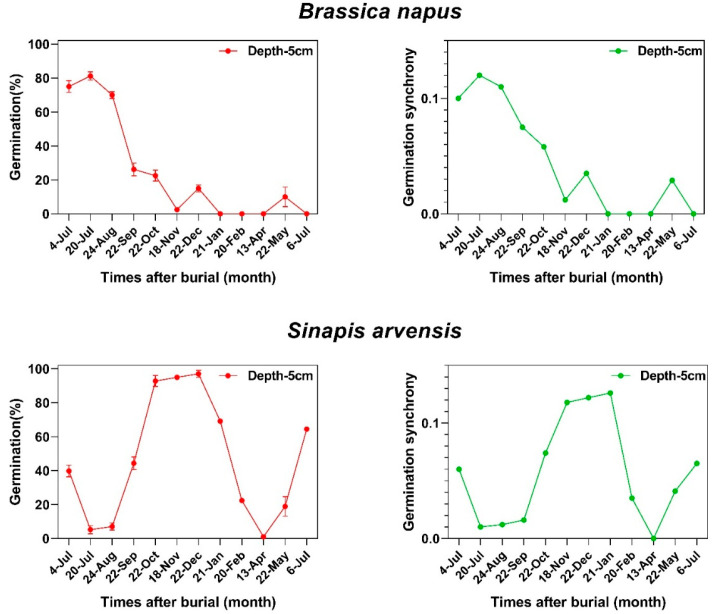
The relationship between germination behavior of two species belonging to Brassicaceae family in the soil seedbank and synchrony patterns.

**Figure 3:**
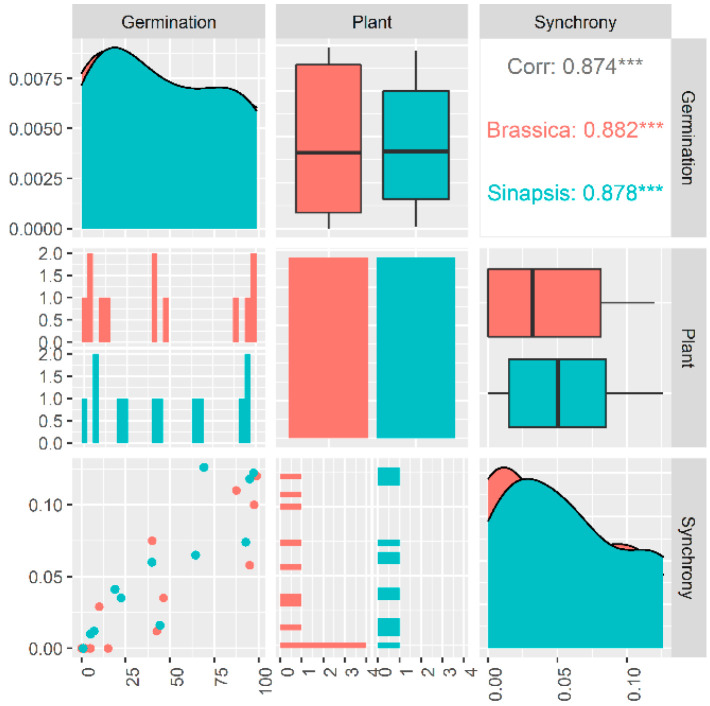
The relationship between germination and germination synchrony of two species belonging to Brassicaceae family. The boxplots, density plots and scatter plot between these traits are indicated. Spearman correlation was used to obtain the boxplots, density plots and scatter plot between these traits. Spearman’s rank correlation coefficient was estimated using Spearman correlation method. *** indicates significance level.

Germination percentage and germination synchrony changed in the same pattern during burial in the soil seedbank in both species (Figure 2). Our data showed that there is a tight link between dormancy cycling and germination synchrony patterns of weed seeds when they are deposited in the soil seedbank (Figure 3) where the seeds of Brassicaceae family show a consistent relationship (r = 0.87; *p* < 0.001) between dormancy level and germination synchrony, suggesting that seeds with lower dormancy levels can germinate in synchrony and this relationship is dynamic across a year (Figure 3). The correlation coefficients between germination and germination synchrony were significant for both species. The boxplots, density plots and scatter plot between these traits are indicated in Figure 3.

### 2.4. Germination Synchrony and Dormancy Type

**Figure 4:**
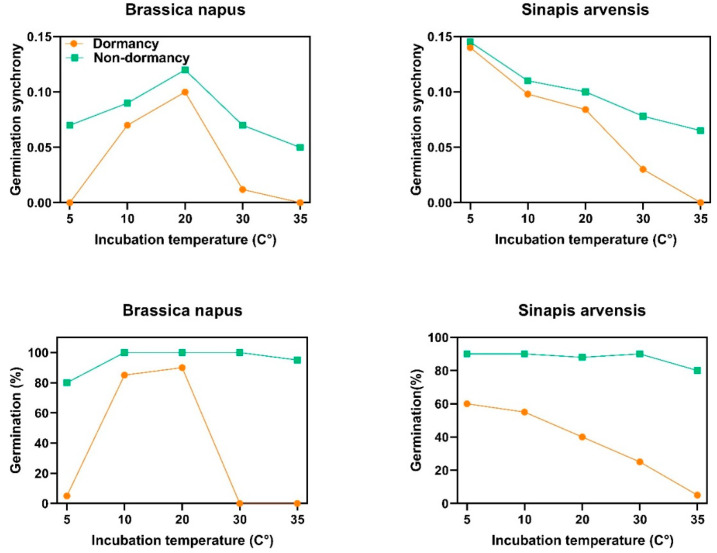
Germination behavior and synchrony patterns of two species of Brassicaceae family at different incubation temperatures. *Brassica napus* and *Sinapis arvensis* have type 3 and 1 non-deep physiological dormancy, respectively. To obtain nondormant seeds, after-ripening was applied as a dormancy-breaking agent.

Dormancy type directly controls the levels of synchrony in seeds with non-deep physiological dormancy. In *B. napus*, which has type 3 non-deep physiological dormancy, dormant seeds can germinate to the highest percentage at intermediate temperature (20 °C). The results showed that germination synchrony is also tightly linked with dormancy type, indicating that dormant seeds of *B. napus* germinated in synchrony at intermediate temperature (20 °C). When seed dormancy was released, the synchrony level increased and seeds gained the ability to germinate synchronously at higher and lower temperatures (Figure 4). In *S. arvensis*, the behavior was different; since this species presents type 1 non-deep physiological dormancy, dormant seeds of this species germinated to the highest percentage at lower temperatures (5 and 10 °C). After becoming nondormant, the germination capacity and synchrony increased to intermediate and higher temperatures (Figure 4).

## 3. Discussion

### 3.1. After-Ripening and Stratification as a Synchronizing Process

Many weed species produce seeds that require dry storage to overcome dormancy [26]. After-ripening defines an effective way in which dormancy can be broken and, initially, refers to the process of gradually releasing dormancy after seed dispersal [27]. Hence, after-ripening has been used to define the growth of embryos undergoing development that exists in some species prior to germination [28] and dormancy release after incubating at cold temperatures [29]. The results showed that after-ripening is a very effective way of releasing non-deep physiological dormancy. Applying after-ripening resulted in changing the dormancy and germination synchrony pattern by regulating germination responses to temperature.

From 1950 onwards, the term after-ripening has been widely used to describe dormancy break during dry storage of seeds [26]. Many researchers have indicated that a period of dry storage at ambient temperature can terminate seed dormancy [30,31,32]. Although much attention has been given to this method, still, more research is required for understanding the underlying mechanisms of this method. Furthermore, after-ripening requirements may be specific to species, and a long duration of after-ripening may act as dormancy-inductive agent, leading to secondary dormancy [26].

### 3.2. Are All Seeds Responsive to After-Ripening?

In considering the effects of after-ripening on germination and dormancy release, the type of dormancy must be incorporated. Based on seed dormancy classification (see dormancy section), physiological dormancy (PD, particularly non-deep PD) is the most common class, with a worldwide distribution [2]. Moreover, most weeds and plants belonging to the Brassicaceae family show non-deep physiological dormancy. As a result, since after-ripening is proven to be one of the most effective methods for breaking seed dormancy, it may have implications for weed scientists and weed management strategies for controlling weed emergence, indicating that remaining weed seeds in the soil seedbank might increase the risk of invasion of weeds, as these plants are responsive to after-ripening and may go through dormancy and synchrony cycling across a year. In seeds showing physiological dormancy, there is a physiological mechanism that prevents germination. Non-deep physiological dormancy is found in many families, including Amaranthaceae, Asteraceae, Brassicaceae, Caryophyllaceae, Euphorbiaceae, Lamiaceae, Myrtaceae, Poaceae, Plantaginaceae, Proteaceae, Scrophulariaceae and Solanaceae [26]. The most important attribute of non-deep physiological dormancy is that different periods of dry storage have the ability to break dormancy; in other words, seeds with non-deep physiological dormancy can after-ripen. After-ripening can increase germination capacity, synchrony, velocity and niche—the range of conditions over which seeds will germinate [16,21,23]. After-ripening can also function as a synchronizing factor (Table 1). We showed that seeds undergoing after-ripening can progressively gain the ability to germinate in concert, suggesting that after-ripening has the potential to synchronize germination, but this phenomenon is highly dependent on the type of dormancy and species.

### 3.3. Stratification

In environments with seasonal variation in temperature patterns, annual and perennial weeds may have seeds that gradually lose dormancy in winter and then germinate in spring, so-called stratification. Stratification defines a cold, wet period that terminates seed dormancy. Stratification is shown as one of the most effective dormancy-breaking agents, which regulates germination synchrony by changing dormancy patterns. In the current study, the application of stratification resulted in lower dormancy. Moreover, the time span required for dormancy loss triggered by stratification is lower than after-ripening, meaning that cold temperature occurring in early fall can be a stimulating factor for the germination of weeds, which is coincident with the cultivation of most crops, leading to the damage caused by weeds [21]. In agricultural settings, stratification, commonly occurring in winter, prevents seeds from germination until spring arrives, suggesting an adaptive strategy delaying germination until ideal conditions exist. Based on the results presented in Table 1, stratification also has the potential for the synchronization of germination.

### 3.4. Seasonal Dormancy Cycling

Seasonal dormancy cycling is a trait commonly found in weed seeds presenting physiological dormancy. The cycling mechanism enables weeds to persist for decades, thereby spreading germination over time until optimal conditions arrive. Regulation of dormancy cycling is environment-dependent, demonstrating that environmental factors, such as temperature and precipitation, may have profound effects on cycling. Furthermore, other life-history events and their related traits, such as germination timing, may be adjusted through the cycling mechanism, elucidating mechanisms behind germination behavior and dormancy patterns. The correspondence between dormancy cycling and germination synchrony might be of great significance when considering weed management, suggesting that germinating in concert may have a weed-suppression role through intraspecific competition. Moreover, the combination of dormancy cycling and differing patterns of synchrony, which shows a consistent trend, may enable weeds to persist, ensuring survival and reproduction.

Germination behavior of seeds experiencing varying environments can be regulated by seasonal cues perceived by seeds and maternal plants, although some seeds may not be responsive to seasonal cues and stay dormant. For example, populations of *B. napus* showing non-deep physiological dormancy went through cycles between dormancy (D)↔conditional dormancy (CD)↔nondormancy (ND) in the soil seedbank [21,23]. Consistent with our theory, Soltani et al. [23] reported that seeds of *B. napus* can go D/CD and become ND and go through the cycles during a year. Germination behavior within a year was tightly linked with seasonal cues. When optimal conditions occur, seeds become nondormant and start germinating to the highest percentage, but when conditions are not suitable (based on environmental factors), seeds become dormant [21]. In this case, there was also an intermediate behavior that shows conditional dormancy, in which seeds only germinate in a narrow range. As a result, conditional dormancy might be an optimal status when environmental conditions are not predictable, implying that seeds having conditional dormancy may adopt an integrated bet-hedging strategy.

### 3.5. Non-Deep Physiological Dormancy as a Determinant of Synchrony

There are six types of non-deep physiological dormancy based on thermal requirement for germination, and different thermal models have been developed to identify dormancy type [16,17,21,33]. Threshold-type responses to temperature, as a descriptor of the characteristics of each species, have been widely used to quantify thermal niche and dormancy type, but here, we established a link between germination responses to temperature and germination synchrony. Based on the thermal time concept, germination occurs when thermal units required for germination of a given fraction (θg) have been accrued. This response is quantified via the function θg = (T − Tb) tg [34]. In this function, T is incubation temperature, Tb is base temperature for germination and tg is time to germination for a given fraction. Seed dormancy can restrict the germination of the Brassicaceae family to a given environmental condition through altering germination thermal niche [21] and subsequently germination synchrony, suggesting that seeds with non-deep physiological dormancy (Type 1, 2 and 3) showing high dormancy may have a narrow thermal niche, while nondormant seeds show a wider thermal niche. Species with non-deep physiological dormancy construct their thermal niche in response to the maternal thermal environment they have experienced. Here, we describe the relationship between non-deep physiological dormancy and synchrony patterns, thereby how non-deep physiological dormancy status adjusts germination by responding to the prevailing temperatures at which seeds start the germination process. Six types of non-deep physiological dormancy were taken into account as follows: Type 1: In this type of non-deep physiological dormancy, dormant seeds only can germinate at low temperatures and germination at higher temperatures will increase as dormancy is terminated, showing that the germination thermal niche of dormant seeds is narrow and limited to low temperatures. Thus, the germination thermal niche widens as dormancy break progresses and, thereby, seeds gain the ability to germinate at higher temperatures. Type 2: Seeds can germinate at high temperatures when dormancy starts to be released; the lowest temperatures at which they will germinate decreases as dormancy break progresses. This type of dormancy is in contrast to Type 1, and germination thermal niche is restricted to higher temperatures. Type 3: Germination occurs only at intermediate temperatures when seeds commence to come out of dormancy, and pervasive temperature ranges at which seeds will germinate both increase and decrease as dormancy break develops, until the widest thermal niche for germination is reached. Type 4: In this type of dormancy, the breadth of the thermal niche is strictly limited to higher temperatures, even when the dormancy is completely broken. Thus, although germination reaches the highest percentage during the dormancy-breaking process, the thermal range over which the seeds can germinate does not change (germination thermal niche is constant over time). Type 5: In contrast to type 4, this type of dormancy limits the thermal niche to low temperatures throughout the dormancy breaking process. Thus, the dormancy-breaking process does not amplify the temperature range over which the seeds can germinate. Type 6: Seeds with Type 6 non-deep physiological dormancy have the widest thermal range possible for the taxon or genotype throughout the dormancy-breaking process. Conditional dormancy is a transitory state between dormancy and nondormancy in which seeds are only able to germinate within a narrow temperature range (limited thermal niche), and as seeds become nondormant, they progressively gain the ability to germinate in a broader temperature range until the widest thermal niche for germination is reached. Conditional dormancy is a strategic mechanism synchronizing germination with favorable conditions by creating a dynamic status altering the breadth of germination thermal niche and enhancing synchrony in response to seasonal cues [2,16,21]. Both *Brassica napus* and *Sinapis arvensis* have non-deep physiological dormancy but the type of dormancy is different. *B. napus* presents type 3 non-deep physiological dormancy, in which dormant seeds only germinate at intermediate temperatures (15 and 20 °C) and when seeds come out from dormancy, they progressively gain the ability to germinate at higher and lower temperatures (i.e., which explains the concept of germination thermal niche influenced by dormancy level). In agreement with the preceding hypothesis on germination capacity, synchrony patterns are also affected by this ecological phenomenon. Germination synchrony of dormant seeds of *B. napus* was higher at intermediate temperatures (15 and 20 °C; Figure 3). Synchrony became enhanced at higher and lower temperatures as dormancy decreased (Figure 3). *S. arvensis* showed a different behavior, as this species exhibits type 1 non-deep physiological dormancy, in which dormant seeds germinate to a higher percentage at lower temperatures, and dormancy loss enables seeds to germinate within a wider temperature range (Figure 3). In this species, germination synchrony of dormant seeds was higher at lower temperatures, which is consistent with dormancy type and level. After releasing dormancy, seeds could better germinate in synchrony at intermediate and higher temperatures (Figure 3).

## 4. Materials and Methods

### 4.1. Germination Synchrony

**Figure 5:**
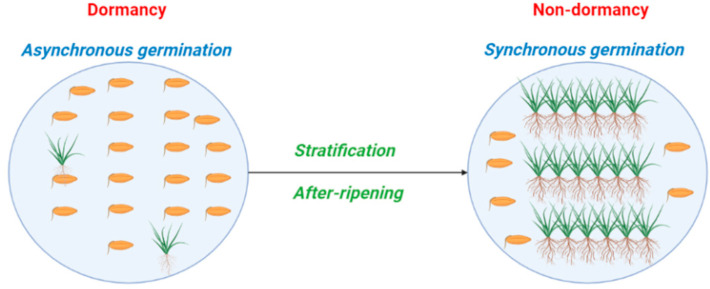
Germination behavior and synchrony patterns of seeds with non-deep physiological dormancy. *Brassica napus* and *Sinapis arvensis* have type 3 and 1 non-deep physiological dormancy, respectively.

We define germination synchrony as temporal propensity of individuals within a population to germinate in concert (Figure 5). The model shows the amount of overlap between germination periods of plants compared to what would be expected to occur randomly under given environmental conditions so populations that are germinating synchronously have more plants germinating at the same time than would be expected to occur randomly. To model germination synchrony, we developed a quantitative framework to provide insight into ecological underpinnings of this trait. Although our conceptual framework can quantify germination synchrony, still, further research is required for understanding the adaptive value of synchrony and the effect of this trait on population dynamics. To explain synchronicity, we present an empirical experiment, in which germination synchrony of two species belonging to Brassicaceae family (*Brassica napus* and *Sinapis arvensis*) exhibiting non-deep physiological dormancy is quantified. To compute germination synchrony, the package germination metrics, which is a set of functions quantifying the time-course of germination via both single-value germination indices and fitted curves, was employed [35,36,37]. Germination asynchrony is computed using RStudio as follows:

Ȇ = −∑f_i_ log2 f_i_ (1)

where Ȇ denotes Synchronization index and fi refers to the relative frequency of germination. According to the concept of model, the lower the values of germination asynchrony, the lower the level of seed dormancy, suggesting that dormant seeds may show higher values of germination asynchrony. This is in contrast to the concept of germination synchrony, indicating that nondormant seeds present a higher value of germination synchrony. Germination synchrony is calculated as follows:

Z = (∑〖C_ni,2_〗)/(C_∑ni,2_) (2)

where Z and C_ni_ denote the time interval, which describes the times that are considered two by two. Here, times are weighted according to the combinatorics, C, of daily counts: C_ni,2_ is the number of possible pair combinations of the ni seeds germinated at time i. C_ni,2_ is calculated as follows:

C_ni,2_ = (n_i_(n_i_ − 1))/2 (3)

The Synchrony Index (Z) used in the current study was developed to assess the extent of overlap between flowering individuals in a population [35]. By developing the idea presented by Aravind et al. [35], the synchrony of a seed with other seeds was included in the same replication. Z equals 1 when germination of all the seeds takes place at the same time and Z equals 0 when at least two seeds can start germinating one at a time. Z shows a number if and only if two seeds finishing the seed germination process at the same time are included. Thus, the value of Z assessments indicates the grade of overlap between seed germination.

### 4.2. Germination Trials

Two sets of experiments in different seedlots of *B. napus* and *S. arvensis* were carried out. In these experiments, we investigated germination phenology of two species exhibiting non-deep physiological dormancy. In studying germination phenology, both dormant and nondormant seeds (after-ripened seeds and seeds undergoing stratification) were taken into consideration to explore how dormancy status underlies synchrony patterns. In both experiments, we immediately examined germination of newly matured seeds in Petri dishes moistened with distilled water (control) at five distinct temperatures (5, 10, 20, 25 and 30 °C) at the time of seed collection. An alternating light and darkness regime (12 h/12 h) was applied to mimic natural conditions occurring in arable lands in Iran.

In the first experiment, to impose after-ripening, we stored the seeds at 20 °C and in darkness (dry condition) for three months [21]. We then tested germination of after-ripened seeds at five incubation temperatures (5, 10, 20, 25 and 30 °C) and alternating light and darkness (12 h/12 h). The aim of this treatment was to see how after-ripening can act as a synchronizing factor by decreasing dormancy level, as suggested by Maleki et al. [25].

In the second experiment, to see how cold stratification can affect germination pattern of *B. napus*, seeds were incubated at 5 °C for five days for potential cold stratification. We then wrapped cold-stratified seeds with aluminum foil and kept them under one constant condition (5 °C). Four replicates of 50 seeds were used in each treatment. Seeds were moistened on filter paper moistened with 6 mL of distilled water in 9 cm-diameter Petri dishes. Water was replenished as needed. The criterion for germination was radicle protrusions ≥ 2 mm recorded on a daily basis when no additional germination was seen for three days. A crush test was used to determine the viability of ungerminated seeds [16].

### 4.3. Burial Experiment

This experiment was performed at the research farm of Aburaihan Campus, University of Tehran, Pakdasht, Iran (35°28′ N, 51°36′ E; 1020 m a.s.l.). Mature seeds of *B. napus,* which were collected on 5 June 2017 from farmer’s field at Gorgan, Iran, were buried at 5 cm depth. Prior to commencing burial experiment, we examined the potential of secondary dormancy induction, as suggested by Weber et al. [36]. The potential of secondary dormancy induction refers to the ability of seeds to enter (re-enter) secondary dormancy in response to environmental cues. In this experiment, we buried 1000 seeds in a nylon bag with a pore size of 10 μm, which was a blend of 10 g of soil. Four replicates of bags were used (in total, 4000 seeds for four replicates). The bags were extracted on a monthly basis, and germination trials were performed with four replications of 50 seeds, as mentioned above.

## 5. Conclusions

In this study, it was shown that the seeds of two Brassicaceae species that have physiological dormancy can be released from dormancy by stratification or after-ripening. Further, a model was presented to describe the synchrony of seed germination, and using this model, it was shown that seeds that come out of dormancy will have more synchrony in germination. The results of this model were also checked in the seeds that were buried in the soil under natural conditions and similar results were obtained. In other words, in natural conditions, these two species come out of dormancy by being exposed to the cold and moisture conditions of winter (stratification) or exposed to the temperature of summer (after-ripening) and their germination becomes synchronized.

Germination synchrony, as a potential trait affecting weed population dynamics, is of great importance when practicing weed management, since this trait can predict the density of emerged seedlings of weeds causing trouble for crops. Having adequate information on synchrony patterns may enable both weed biologists and agronomists to be well prepared for the timing and density of weeds and finding the best methods for suppressing these troublesome plant species. Dormancy level and type underlie changes in germination synchrony patterns, and dormancy-breaking agents (i.e., after-ripening and stratification) have considerable effects on regulating synchrony level. Conditional dormancy directly controls germination behavior through altering the level of synchrony. Germination synchrony may be an adaptive trait since this trait corresponds well with dormancy level, type, germination niche and the mechanism timing germination episodes with favorable conditions. Evidence for eco-evolutionary responses and the role of adaptive traits in producing the responses has been obtained in recent years. However, a primary outcome is that weeds tend to maintain seed dormancy as a fundamental trait, to provide insurance against uncertainty, implying that all adaptive traits and strategies correspond well with dormancy state. Seed dormancy underlies all changes in adaptive strategies. Synchronization, for example, as a potential adaptive process adjusting germination phenology and modifying the conditions species are likely to encounter, is directly influenced by dormancy status. Compared to other process-based models and parameters used to quantify germination uniformity and synchrony, the germination synchrony model can simply germination pattern estimates, degree of dormancy and dormancy cycling during the year, suggesting a useful method for quantification of germination strategies.

## Figures and Tables

**Table 1:** Synchrony pattern of dormant and nondormant seeds.

Species	Mature Seed-Dormant Seed		After-Ripening-Non Dormant	
	Asynchrony	Synchrony	Asynchrony	Synchrony
**Experiment 1**				
*Brassica napus*	3.14 ± 0.016	0.089 ± 0.002	2.57 ± 0.042	0.116 ± 0.007
*Sinapis arvensis*	2.40 ± 0.014	0.054 ± 0001	2.84 ± 0.072	0.132 ± 0.002
**Experiment 2**	**Mature seed-Dormant seed**		**Stratification-non dormant**	
	**Asynchrony**	**Synchrony**	**Asynchrony**	**Synchrony**
*Brassica napus*	2.75 ± 0.022	0.023 ± 0.002	1.95 ± 0.062	0.115 ± 0.002
*Sinapis arvensis*	2.25 ± 0.012	0.038 ± 0.004	2.00 ± 0.009	0.140 ± 0.0029

## Data Availability

Not applicable.
